# Synthesis and Characterization of Hybrid Core-Shell Fe3O4/SiO2 Nanoparticles for Biomedical Applications

**Published:** 2017

**Authors:** I. V. Zelepukin, V. O. Shipunova, A. B. Mirkasymov, P. I. Nikitin, M. P. Nikitin, S. M. Deyev

**Affiliations:** Shemyakin-Ovchinnikov Institute of Bioorganic Chemistry, Russian Academy of Sciences, Miklukho- Maklaya Str. 16/10, Moscow, 117997, Russia; Moscow Institute of Physics and Technology, Institutskiy Per. 9, Moscow Region, Dolgoprudny, 141700 , Russia; National Research Nuclear University MEPhI, Kashirskoe shosse 31, Moscow, 115409, Russia; Prokhorov General Physics Institute, Russian Academy of Sciences, Vavilov Str. 38, Moscow, 119991 , Russia; Lobachevsky State University of Nizhny Novgorod, Gagarina prospekt 23, Nizhny Novgorod, 603950 , Russia

**Keywords:** magnetic nanoparticles, surface modification, magnetic detection, silicium dioxide, cell labeling

## Abstract

The creation of markers that provide both visual and quantitative information is of considerable importance for the mapping of tissue macrophages and other cells. We synthesized magnetic and magneto-fluorescent nanomarkers for the labeling of cells which can be detected with high sensitivity by the magnetic particle quantification (MPQ) technique. For stabilization under physiological conditions, the markers were coated with a dense silica shell. In this case, the size and zeta-potential of nanoparticles were controlled by a modified Stober reaction. Also, we developed a novel facile two-step synthesis of carboxylic acid-functionalized magnetic SiO_2_ nanoparticles, with a carboxyl polymer shell forming on the nanoparticles before the initiation of the Stober reaction. We extensively characterized the nanomarkers by transmission electron microscopy, electron microdiffraction, and dynamic and electrophoretic light scattering. We also studied the nanoparticle cellular uptake by various eukaryotic cell lines.

## INTRODUCTION

There is growing interest at the moment in the use of nanoparticles as theranostic objects (agents that combine diagnostic and therapeutic functions on one single platform) [1-3] and in the development of nanocomplexes capable of performing a therapeutic function or binding to cells only in response to certain signals from the body or to the absence of such signals [4], or external stimuli [5]. For an early diagnosis of diseases and the monitoring of ongoing therapy, it is important to be able to visualize the distribution of nanoagents in the body by means of various markers.

Many magnetic nanoparticles are superparamagnetic, which makes them detectable by magnetic resonance imaging [6], MPI visualization [7], ferromagnetic resonance [8], giant magnetic resistance [9], etc. [10-12]. Of particular interest is the detection of nonlinear magnetic materials, which is based on the exposure of a sample to a magnetic field at two frequencies and the monitoring of the response at combinatorial frequencies of the applied field (MPQ detection) [13]. This method enables highly sensitive and quantitative detection of superparamagnetic nanoparticles in a wide range of concentrations, in particular non-invasively in a living organism, which opens up broad prospects for their use in biomedicine.

Nonstabilized magnetic nanoparticles do not have colloidal stability under physiological conditions, and they are susceptible to oxidation, which can decrease their detection limit [14]. An effective dense coating that can protect magnetic particles from oxidation and aggregation is a silica shell. Such a coating is highly stable and inert, and its surface can be modified by the desired functional groups. In addition, the mesoporous silica structure is used to deliver therapeutic agents and genetic vectors [15].

Silica-containing nanoparticles are often synthesized using the Stober method. This is a simple and convenient one-step method that avoids surfactants or toxic organic solvents, and a relatively low rate of inorganic layer formation enables one to control the resulting nanoparticle’s size [16].

We synthesized magnetic and magneto-fluorescent markers coated with a silica shell. The particle surface was functionalized with amino and carboxyl groups to ensure use of these markers for conjugation with other nanoagents, proteins, and targeting moieties. We also proposed a method for coating magnetic particles with silica without the need for further modification of the surface by functional groups. We studied the synthesized nanoparticles by transmission electron microscopy, electron microdiffraction, and dynamic and electrophoretic light scattering, and we measured the detection limit of the nanoparticles as magnetic markers for biomedical research. We demonstrated effective quantitative and optical labeling of various eukaryotic cells by the nanoparticles and found a relatively low cytotoxicity of the markers at the tested concentrations.

The produced markers are promising for use *in vivo*: e.g., to identify tissue macrophages and determine their activity for the diagnosis of atherosclerosis, cancers, myocardial infarction, and other human diseases [17, 18].

## EXPERIMENTAL

In the study, we used iron (II) chloride tetrahydrate, iron (III) chloride hexahydrate, tetraethyl orthosilicate (TEOS), (3-aminopropyl)triethoxysilane, tris(2,2’- bipyridyl)ruthenium (II) chloride hexahydrate, succinic anhydride, carboxymethyl dextran sodium salt, *L*-glutamine, dye Hoechst 33342 (Sigma-Aldrich), aqueous ammonia, nitric acid, trisodium citrate dihydrate, isopropyl alcohol, ethyl alcohol, dimethyl sulfoxide (Chimmed), ninhydrin, MTT solution (Dia-m), dry methyl alcohol (Merck), Concanavalin A (lectin from *Canavalia ensiformis*) (Vector Laboratories), phosphate buffered saline (PBS) pH 7.4, carbonate buffer pH 9, McCoy’s 5A medium (Life Technologies), fetal bovine serum (FBS) (HyClone), and BT-474, SK-BR-3 (human mammary gland), HEK 293T (human embryonic kidney), and CHO (Chinese hamster ovary) cell lines. For magnetic separation, a permanent cylindrical Neodymium Iron Boron magnet D 25 × 10 mm (Ningbo Ketian Magnet Co.) was used.

**Synthesis of magnetite nanoparticles**

A mixture containing 2.9 mmol of FeCl_2_•4H_2_O, 10.1 mmol of FeCl_3_•6H_2_O, and 40 mL of distilled water was added with 5 mL of 30% NH_4_OH under constant stirring. The solution was kept at 80 °C for 2 h. The resulting particles were treated with a 2M HNO_3_ solution and then repeatedly washed with distilled water by magnetic separation on a 25 mm Neodymium Iron Boron magnet. The particles not attracted to the magnet were sequentially collected for 15 min, thereby forming different fractions of magnetic particles. The first two fractions had low pH values, which led to a rapid degradation of particles. Magnetic nanoparticles of the third fraction were used in the experiments.

**Coating of nanoparticles with a silica shell**

To stabilize magnetic nanoparticles under the reaction conditions, the particles were pre-coated with a citrate anion by adding trisodium citrate (a concentration of 25 g/L) to a colloidal solution of magnetic particles. Alternatively, the particles were coated with a polymeric carboxymethyl dextran layer. For this, carboxymethyl dextran was added (to get a final concentration of 50 g/L) to a colloidal nanoparticle solution under heating to 80°C. After preliminary stabilization, the magnetic particles were washed three times with distilled water.

Fifty microliters of magnetic particles was added to 1 mL of alcohol. The reaction mixture pH was adjusted to 9, and then 10 – 200 μL of TEOS was added. After 1 day, the nanoparticles were washed by centrifugation with distilled water.

**Functionalization of the silica nanoparticle surface**

A 1% solution of (3-aminopropyl)triethoxysilane in ethanol was added to the synthesized particles, which led to exposure of the primary amino groups on their surface. The particles were then washed twice with ethanol. Further, the amino groups were modified into carboxyl groups by the addition of succinic anhydride in a carbonate buffer (pH 9) to a concentration of 4 g/L. After 3 h, the particles were washed from the reaction products with distilled water.

**Preparation of magneto-luminescent silica nanoparticles**

Magneto-luminescent nanoparticles were prepared analogously to the magnetic particles coated with silica in ethanol by adding 0.03 mg of tris(2,2’-bipyridyl)ruthenium (II) chloride hexahydrate to 1 mL of the reaction mixture 5 min after the start of TEOS hydrolysis. After the synthesis, the nanoparticles were stored in the dark at +4 °C.

**Characterization of particles**

The hydrodynamic nanoparticle size and zeta potential were determined by dynamic light scattering and electrophoretic light scattering using a Zetasizer Nano ZS analyzer (Malvern Instruments Ltd.). We used the mean particle size and the mean zeta potential value. To measure the zeta potential, the particles were transferred to PBS, pH 7.4, before measurement.

The morphology of the nanoparticles was examined using a JEM-2100 transmission electron microscope (JEOL Ltd.) with an accelerating voltage of 200 kV. The nanoparticle samples were applied to a carbon-coated copper grid and then dried in air.

The phase composition of the particles was determined by the electron microdiffraction method.

The magnetic signal of iron oxide markers was determined by MPQ detection of nonlinear magnetic materials [13]. For measurement, 20 μL of the nanoparticle sample in the cylindrical tube was placed into the coil of the MPQ reader.

Fluorescence and absorption spectra were acquired using an Infinite M1000PRO Microplate reader (Tecan Group Ltd.).

**Cell labeling with nanoparticles**

Cells of the BT-474, SK-BR-3, HEK 293T, and CHO lines were cultured in a McCoy’s 5A medium supplemented with 10% heat-inactivated fetal bovine serum and 2 mM *L*-glutamine at +37°C in a humidified atmosphere with 5% CO_2_. The cells were passaged 2 to 3 times a week at 80–90% confluence. The cells removed from the culture plastic surface (0.7 × 10^6^) were washed twice with PBS, incubated with nanoparticles at a concentration of 0.01 g/L at room temperature for 2 h, and washed from unbound particles under constant stirring. The number of cell-bound particles was determined by MPQ-cytometry [18].

**Cell viability assay**

Nanoparticle cytotoxicity was assessed using an MTT test. Cells were seeded on a 96-well plate, 104 cells/well into 200 μL of McCoy’s 5A medium with 10% FBS. The cells were cultured at 37°C in a CO_2_ incubator overnight. Then, the medium was removed and the cells were sterilely added with a serum-free medium (negative control) and a serum-free medium containing the tested particles at various concentrations at a volume of 100 μL per well. The cells were incubated at room temperature for 2 h, then washed with the serum-free medium, added with McCoy’s 5A medium containing 10% FBS, and incubated in a CO_2_ incubator (24 h, 37°C). The medium was then shaken off, and the cells were washed once with the medium. After this, 100 μL of a MTT solution (0.5 g/L in McCoy’s 5A) was added per well and incubated at 37 °C in a 5% CO_2_ atmosphere for 1 h. Then, the MTT solution was removed, 100 μL of dimethyl sulfoxide was added per well, and the plate was shaken until complete dissolution of formazan crystals. The solution absorbance in each well was measured using an Infinite M1000PRO Microplate reader - (Tecan Group Ltd.) at a wavelength of λ=540 nm.

**Fluorescence microscopy**

Cells were plated into a 96-well plate, 104 cells/well in 200 μL of McCoy’s 5A medium with 10% FBS. After culturing at 37°C in a CO_2_ incubator overnight, the tested particles were sterilely added to the cells and the cells were incubated at room temperature for 2 h, washed with serum-free medium, added with McCoy’s 5A medium with 10% FBS, and incubated in a CO_2_ incubator at 37°C for 24 h. Cell nuclei were stained with the Hoechst 33342 dye at room temperature for 10 min and then washed three times with PBS. Cell samples were analyzed with a Leica DMI 6000B inverted fluorescent microscope (Leica Microsystems) in transmitted light and fluorescence channels corresponding to nanoparticle fluorescence (excitation at 545/30; emission at 610/75) and Hoechst 33342 dye fluorescence (excitation at 360/40; emission at 470/40).

## RESULTS AND DISCUSSION

**Fig. 1:**
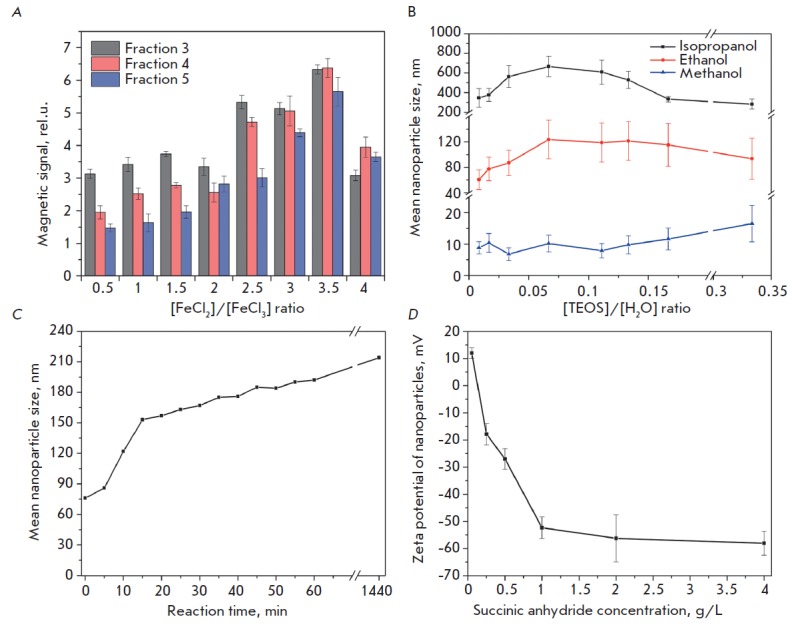
Control of the physico-chemical properties of magnetic silica nanoparticles: (*A*) the dependence of the normalized nanoparticle magnetic signal on the ratio of iron salts; (*B*) the dependence of the hydrodynamic particle size on the [TEOS]/[H_2_O] ratio in the Stober process for various solvents; (*C*) the dependence of the hydrodynamic particle size on time in the Stober reaction; (*D*) the effect of the succinic anhydride concentration in the reaction mixture on the particle zeta potential. Error bars indicate the standard deviation from the mean particle size.

Magnetite nanoparticles were synthesized by co-precipitation of iron (II) and (III) chlorides under alkaline conditions. The synthesis was optimized to produce magnetic markers with a minimum detection limit. Because many iron oxyhydroxides produced in the reaction were not superparamagnetic and reduced the detectable magnetic signal of the entire nanoparticle sample [19], it was very important to determine the optimum ratio of iron salts in the reaction mixture. The maximum, normalized signal of particles was found to occur at a salt ratio of [FeCl_2_]/[FeCl_3_]=1/3.5. In this case, the maximum magnetic signal was observed in the third and fourth fractions of nanoparticles (*Fig. 1A*). The detection limit of these nanoparticles determined with MPQ was found to be 2.7 ng in 20 μL of solution.

Then, the nanoparticles were coated with a silica shell. The zeta potential of magnetic nanoparticles at pH 9 was near zero, which led to their aggregation under the reaction conditions. The agglomerates that formed at high pH lost colloidal stability. Therefore, it was necessary to modify the particles before the synthesis of the silica coating. For this purpose, as in [20], we used a citrate coating (hereinafter, these particles are designated as m-cit). In this case, the zeta potential of the nanoparticles became strongly negative, and the particles remained stable over a wide range of pH values. For the first time, a polymeric carboxymethyl dextran was used as an alternative intermediate coating. The magnetic nanoparticles coated with carboxymethyl dextran (hereinafter m-CMD) were stable under the reaction conditions. In addition, the polymer bounded several magnetite particles together, which resulted in polymer-coated particles with a high content of magnetic nuclei, and, hence, a lower detection limit. Then, hydrolysis of tetraethyl orthosilicate with polycondensation of the reaction products on the magnetite surface was performed.

Synthesis of silica nanoparticles lacking a magnetic core was used as a model system for exploring the main dependencies of the synthesis process. We studied the effect of parameters such as the solvent type, [H_2_O]/ [TEOS] ratio, and the reaction pH on the size of the resulting SiO_2_ nanoparticles.

Increase in the carbon chain length in the used alcohol was found to result in a substantial increase in the size of the synthesized particles. The mean silica particle size was ~ 10 nm in methanol, 100 nm in ethanol, and 500 nm in isopropanol. Solvents with a longer carbon chain are hydrophobic, which is not compatible with the standard Stober reaction. A change in the [H_2_O]/[TEOS] ratio in the reaction enabled a more accurate control of the silica particle size (*Fig. 1B*). The dependence of the hydrodynamic particle size on the reagent ratio had a characteristic profile with a pronounced maximum, which was probably related to a decrease of tetraethyl orthosilicate or water quantities due to the reaction within the tested concentration range. The Stober process proceeded at alkaline pH, and increase in the pH significantly accelerated the reaction, which adversely affected the particle size dispersion. Most of the experiments were carried out at pH 9, with the particle synthesis time being approximately 1 h (*Fig. 1C*).

We synthesized both magnetic and magnetic-luminescent silica-coated nanoparticles. The growth of magnetic silica particles depended on the reaction conditions in the same pattern as that of SiO_2_ particles. In particular, the [TEOS]/[H_2_O] ratio in the synthesis of m-cit-SiO_2_ influenced the nanoparticle size in the same pattern as was previously determined for SiO_2_ nanoparticles.

The use of methanol or ethanol as a solvent resulted in magnetic particles with mean sizes ranging from 50 to 80 nm and 100 to 200 nm, respectively. In isopropanol, citrate-coated magnetic particles aggregated: therefore, before starting the reaction, the particles were first coated with a thin SiO_2_ layer in methanol and then used as nucleation centers in the next step of the Stober process in isopropanol. This procedure resulted in particles of 300–500 nm in size.

Using carboxymethyl dextran as an intermediate coating, we obtained nanoparticles with mean sizes of 200 ± 60 nm, with the mean size of initial m-CMD particles being 44 ± 12 nm. Nanoparticles of 200 ± 60 nm in size were used twice as “seeds” in the Stober reaction in isopropanol to produce particles of 764 ± 187 nm in size. The use of the described particles as nucleation centers in a multistage variant of the Stober reaction resulted in large-size particles that were not appropriate for *in vivo* experiments [21] but interesting for *ex vivo* and *in vitro* diagnostics.

**Fig. 2:**
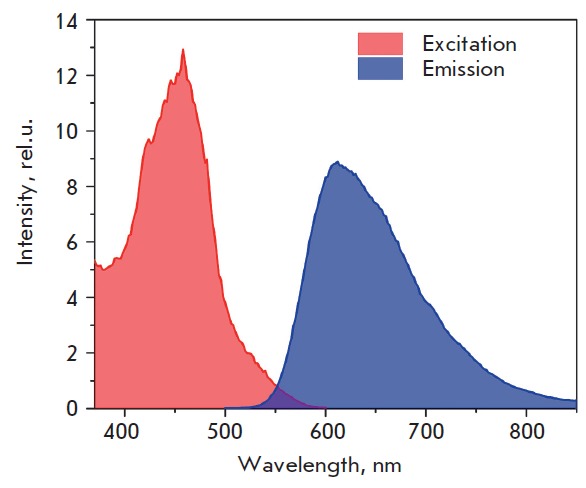
Excitation and emission spectra of luminescent magnetic silica nanoparticles.

To synthesize fluorescent nanoparticles, we, as in [22], added [Ru(bipy)3]Cl2 to the reaction mixture 5 min after starting the reaction to avoid the aggregation of magnetic nuclei through an increase in the ionic strength of the solution. Tris(2,2’-bipyridyl)ruthenium (II) was incorporated into the forming amorphous silica lattice, which induced fluorescent properties in the particles. The excitation and emission spectra of the particles are shown in *Fig. 2*. The particles retained their colloidal stability and ability to fluoresce for at least 1 year.

For various biological applications, conjugation of nanoparticles with proteins or other objects is often necessary. Bioconjugation chemistry allows one to couple objects of different nature via certain functional groups. One of the most convenient techniques is carbodiimide conjugation of a carboxyl group to a primary amino group, resulting in the formation of a stable peptide bond [23]. The synthesized m-cit-SiO_2_ nanoparticles initially exposed surface hydroxyl groups, so we performed a two-step modification of their surface to obtain carboxyl groups. First, the surface of SiO_2_ particles was treated with (3-aminopropyl)triethoxysilane. The presence of surface amino groups was proved by a change in the color of a nanoparticle solution upon its interaction with a 5% ninhydrin solution and also by a change in the mean particle zeta potential from –36 to +12 mV. After that, the particles were treated with succinic anhydride and surface amino groups were converted to carboxyl groups by the ring opening reaction. The logarithmic dependence of the resulting zeta potential of particles on the succinic anhydride concentration in the mixture enabled the production of particles with different zeta potentials ranging from +12 to –58 mV (*Fig. 1D*).

Surface-exposed amino groups cause particle aggregation, but after the second stage of modification, the hydrodynamic particle size becomes equal to that of the original particles without aggregate formation, which made it possible to use the method in producing colloidally stable solutions of differently charged magnetic silica particles.

It should be noted that the use of carboxymethyl dextran for intermediate stabilization of magnetite eliminates the need for an additional modification of the surface of magnetic silica particles, because the carbohydrate polymer with carboxyl groups occurs on the surface immediately after the synthesis. The presence of dextran on the surface was confirmed by sedimentation of particles in the presence of Concanavalin A that bound carbohydrates and, consequently, the polysaccharide on the particles surface [24]. Thus, the use of carboxymethyl dextran accelerates the synthesis and provides, immediately after the Stober reaction, markers ready for conjugation with proteins.

**Fig. 3:**
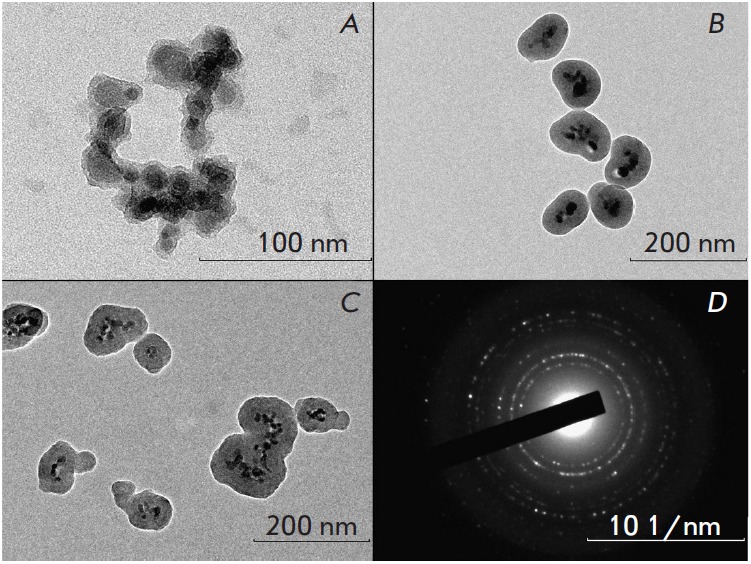
Electron micrographs showing the typical architectures of magnetic silica nanoparticles. Nanoparticles were synthesized in methanol (*A*) or ethanol (*B*) through intermediate stabilization with citrate and in ethanol through intermediate coating with carboxymethyl dextran (*C*). (*D*) shows a microdiffraction pattern of magnetic nanomarkers.

The morphology of m-cit-SiO_2_ magnetic silica nanoparticles with surface carboxyl groups, synthesized in ethanol and methanol, and m-CMD-SiO_2_ particles synthesized in methanol was studied by transmission electron microscopy and electron microdiffraction (*Fig. 3*).

The obtained electron micrographs revealed that all synthesized nanoparticles were multinuclear structures containing 2 to 30 magnetite nuclei and having a solid silica shell with a thickness of 2 (*Fig. 3A*) to 30 nm (*Fig. 3C*). The m-CMD-SiO_2_ particles had more iron oxide nuclei than m-cit-SiO_2_ ones, on average, and had a detection limit determined with MPQ of 2.7 ng in 20 μL of solution, which is comparable or superior to many methods widely used in magnetometry. It should be noted that the nanoparticle’s size determined from image analysis correlates with the dynamic light scattering data. An analysis of the diffraction spectrum of the magnetic nuclei demonstrated that they consisted of magnetite Fe_3_O_4_, the crystallographic Fd3m space group (cubic system), with the main interplanar distances in the crystal being 0.49, 0.29, 0.25, 0.21, 0.17, and 0.15 nm. The strongest line was 0.25 nm.

The synthesis of magnetic nanoparticles capable of effectively interacting with the surface of living cells is important in such areas as MRI monitoring of stem cells, magnetic tissue engineering, magnetofection of eukaryotic cells, and some others. Previous studies have demonstrated that one of the important characteristics that determine the interaction of particles with proteins and cells is the zeta potential [25]. While a positive charge on a particle’s surface leads to a more active adsorption of proteins, a strong negative charge significantly increases the efficiency of cellular uptake by particles [25].

**Fig. 4:**
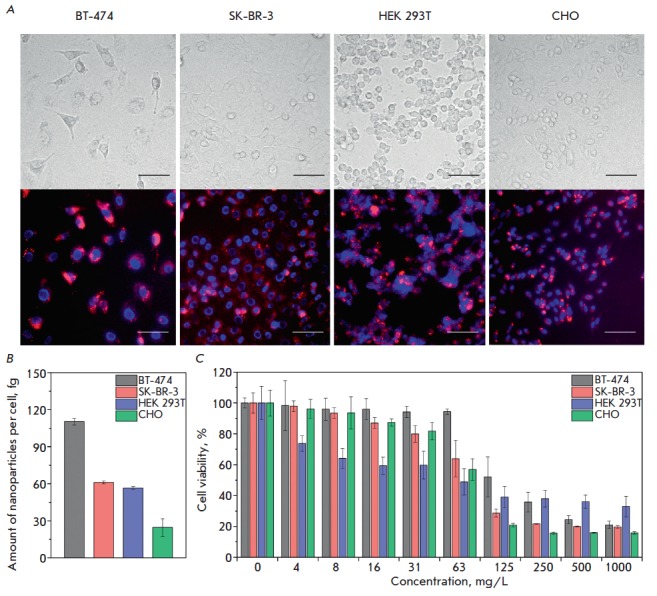
Labeling of eukaryotic cells by magneto-fluorescent m-CMD-SiO_2_ nanoparticles. (*A*) Fluorescence microscopy: visualization of different cells with m-CMD-SiO_2_ nanoparticles. Top panel: transmitted light images; bottom panel: overlaying of fluorescence light images of nanoparticles (excitation at 545/30, emission at 610/75) and the nuclear dye Hoechst 33342 (excitation at 360/40, emission at 470/40). Scale bars – 75 μm. (*B*) Cellular uptake of m-CMD-SiO_2_ nanoparticles measured by MPQ-cytometry. (*C*) Cytotoxicity of m-CMD-SiO_2_ nanoparticles.

In this paper, we have demonstrated effective labeling of eukaryotic cells from different tissues and species by negatively charged m-CMD-SiO_2_-particles. The cell lines BT-474, SK-BR-3, HEK 293T, and CHO were incubated with nanoparticles and washed from unbound particles for further analysis. Using fluorescence microscopy, we found that these particles were able to effectively visualize eukaryotic cells (*Fig. 4A*), with the cell membrane integrity being preserved. Upon interaction with the cell membrane, the particles, despite their high colloidal stability, tended to form bright and visually detectable large conglomerates.

Nanoparticles used as markers of the cell surface should possess high biocompatibility. Therefore, we compared the cytotoxicity of the particles in the MTT test. At the nanoparticle concentration used for cell visualization, namely 0.01 g/L, more than 85% of the cells (except the HEK 293T line) retained their viability (*Fig. 4C*). In this case, IC_50_ of m-CMD-SiO_2_ particles for all four cell lines was in the range of 63–125 mg/L, which indicates their low cytotoxicity, comparable to that of other magnetic nanoparticles used *in vivo* [26].

It is interesting to note that ruthenium (II)-based fluorescent compounds can be considered for use as chemotherapeutic agents [27]. But in our case, the presence of ruthenium (II) did not significantly affect particle toxicity, probably due to the strong fixation of ruthenium in a silica shell.

The physico-chemical properties of these particles, such as fluorescence and magnetism, as well as the opportunity of their effective modification by biomolecules, make the particles very promising for diagnostic purposes. These nanoparticles can be simultaneously visualized and quantified in explored sites of their uptake. For example, we used MPQ-cytometry to quantify interactions between m-CMD-SiO_2_ nanoparticles and the mentioned cell lines and revealed statistically different uptakes of the nanoparticles in different cells, expressed in the mass content of particles per cell (BT-474: 110.4 ± 1.3; SK-BR-3: 61.1 ± 1.2; HEK 293T: 56.6 ± 1.3; CHO: 24.6 ± 7.2 fg/cell). It should be noted that even a smaller amount of magnetic particles associated with cells is sufficient not only for *in vitro *imaging of cells, but also for tracking cells in a living organism [28].

Therefore, we synthesized magnetic and magneto-fluorescent particles with the desired features: magnetism, fluorescence, and controlled surface properties. These particles were effectively used for the labeling of eukaryotic cells, with the integrity and viability of the cells being preserved. The particles can be detected with high sensitivity using the original method for the detection of nonlinear magnetic materials. The synthesized SiO_2_-coated nanoparticles may be further linked to various biopolymer molecules [29] and used for tar geted drug delivery. In addition, they are promising cell surface markers for such biological and biomedical applications as tissue engineering, various immunoassays, as well as different nanobiotechnology aspects where highly efficient labeling of cells with magnetic particles is necessary in order to further affect the resulting cell-nanoparticle complexes [30].

## Abbreviations

MPI: magnetic particle imaging
MPQ: magnetic particle quantification
TEOS: tetraethyl orthosilicate
m-cit: citrate-coated magnetic particles
m-CMD: carboxymethyl dextran-coated magnetic particles
m-cit-SiO2: magnetic particles coated by SiO2 via an intermediate citrate coating
m-CMD-SiO2: magnetic particles coated by SiO2 via an intermediate carboxymethyl dextran coating
